# Genetic Variant Analyses Identify Novel Candidate Autism Risk Genes from a Highly Consanguineous Cohort of 104 Families from Oman

**DOI:** 10.3390/ijms252413700

**Published:** 2024-12-21

**Authors:** Vijay Gupta, Afif Ben-Mahmoud, Ahmed B. Idris, Jouke-Jan Hottenga, Wesal Habbab, Abeer Alsayegh, Hyung-Goo Kim, Watfa AL-Mamari, Lawrence W. Stanton

**Affiliations:** 1 Neurological Disorder Research Center, Qatar Biomedical Research Institute (QBRI), Hamad Bin Khalifa University (HBKU), Qatar Foundation, Doha P.O. Box 5825, Qatar; vgupta@hbku.edu.qa (V.G.); abenmahmoud@hbku.edu.qa (A.B.-M.); jjhottenga@gmail.com (J.-J.H.); whabbab@hbku.edu.qa (W.H.); hk1047@rwjms.rutgers.edu (H.-G.K.); 2 Developmental Paediatric Unit, Sultan Qaboos University Hospital, Sultan Qaboos University, Muscat 123, Oman; ahmed30411@gmail.com; 3 Department of Biological Psychology, Vrije Universiteit Amsterdam, 1081 BT Amsterdam, The Netherlands; 4 Genomics Department, Sultan Qaboos Comprehensive Cancer Care and Research Center, University Medical City, Muscat 123, Oman; abeer.alsayegh@gmail.com; 5 Department of Neurosurgery, Robert Wood Johnson Medical School, Rutgers, The State University of New Jersey, Piscataway, NJ 08854, USA; 6 College of Health & Life Sciences, Hamad Bin Khalifa University (HBKU), Qatar Foundation, Doha P.O. Box 5825, Qatar

**Keywords:** autism spectrum disorder (ASD), genome sequencing (GS), variant of unknown significance (VUS), candidate ASD risk genes

## Abstract

Deficits in social communication, restricted interests, and repetitive behaviours are hallmarks of autism spectrum disorder (ASD). Despite high genetic heritability, the majority of clinically diagnosed ASD cases have unknown genetic origins. We performed genome sequencing on mothers, fathers, and affected individuals from 104 families with ASD in Oman, a Middle Eastern country underrepresented in international genetic studies. This approach identified 48 novel candidate genes significantly associated with ASD in Oman. In particular, 35 of these genes have been previously implicated in neurodevelopmental disorders (NDDs) in other populations, underscoring the conserved genetic basis of ASD across ethnicities. Genetic variants within these candidate genes that would impact the encoded protein included 1 insertion, 4 frameshift, 6 splicing, 12 nonsense, and 67 missense changes. Notably, 61% of the SNVs were homozygous, suggesting a prominent recessive genetic architecture for ASD in this unique population. The scarcity of genetic studies on ASD in the Arabian Peninsula has impeded the understanding of the unique genetic landscape of ASD in this region. These findings help bridge this knowledge gap and provide valuable insights into the complex genetic basis of ASD in Oman.

## 1. Introduction

ASD is a neurodevelopmental condition characterized by difficulties in social communication and interaction, often accompanied by restricted and repetitive behaviours, interests, or activities [1,2]. Based on the Diagnostic and Statistical Manual of Mental Disorders, Fifth Edition (DSM-5), ASD symptoms vary in severity and include challenges in social interactions, such as difficulty with eye contact, understanding social cues, or engaging in reciprocal conversations [3,4,5]. Globally, ASD affects approximately 1% of children, with a pronounced sex disparity, as males are diagnosed about four times more frequently than females [3,5]. Individuals with ASD frequently display co-occurring mental, neurological, or physical comorbidities, including intellectual disability, seizures, sleep disorders, craniofacial anomalies, and gastrointestinal problems, suggesting complex genetic etiologies [6,7,8,9].

Populations in the Middle East and North Africa (MENA) region exhibit high genetic diversity and are underrepresented in genetic studies, especially in autism research [10]. This diversity stems from factors like historical migration patterns, tribal ancestry, and high consanguinity rates, ranging from 20% to 50% across the Middle East, particularly in the Arab Gulf countries [10,11,12,13,14]. Such high consanguinity rates are associated with a higher prevalence of genetic disorders and birth defects [11,12,13,14,15,16,17]. Distinctive genomic patterns in MENA populations, particularly in Saudi Arabia, the UAE, Oman, and Qatar, provide unique insights into genetic factors underlying both common and rare diseases. These patterns may also reveal specific ASD risk genes that have remained undetected in other populations [15,18,19,20,21,22].

ASD research in the Middle East has grown significantly in recent years. Notably, a study in Qatar reported an ASD prevalence rate of 1 in 87 children aged 6 and 11, aligning with global rates and challenging previous assumptions of lower prevalence in this region [23]. This has raised awareness and driven further ASD research and support in the Middle East [18,24,25,26,27]. In 2019, a pioneering study found ASD prevalence in Omani children to be 15 times higher than previous estimates, highlighting the importance of proper diagnostic methods and enhanced ASD awareness [28].

ASD is associated with highly heterogeneous genetic mutations affecting various biological processes and pathways [29,30,31], including synaptic plasticity, chromatin remodeling, gene transcription, and protein degradation [32,33,34,35,36]. These mutations include single nucleotide variants (SNVs), rare copy number variations (CNVs), and chromosomal structural changes [33,35,37,38]. Traditional positional cloning and linkage analysis have identified only a limited number of ASD genes, hampered by the scarcity of families with multiple affected members and patients with chromosomal anomalies. However, the advent of next-generation sequencing (NGS) technologies such as exome sequencing (ES) and genome sequencing (GS) have revolutionized gene discovery by generating massive genomic datasets that greatly expand the scope of genetic research [19,30,31,39,40,41,42,43,44,45,46,47,48,49,50,51,52]. Simultaneously, large-cohort analyses have become increasingly prominent, revealing critical associations between genetic variants and human diseases across diverse populations [29,30,31,39,42]. Together, these innovations are advancing precision medicine, enabling researchers to more accurately map genetic risk factors and develop tailored interventions at the individual level.

Extensive studies highlight de novo variants (DNVs) as major contributors to ASD genetics, while recessive inherited variants appear less common, with estimates of 1.1% in Autism Speak’s MSSNG database and 0.3% in the Simons Simplex Collection [53]. However, recessive variants are increasingly recognized as contributors to ASD, particularly in consanguineous populations, where it may account for ~5% of cases [54,55]. Intriguingly, studies of populations with high consanguinity report up to 39% of ASD cases associated with recessive inheritance, underscoring the importance of this genetic pattern in these populations [56]. Examining populations with high consanguinity is essential to comprehensively understand ASD’s genetic architecture, including both de novo and recessive inheritance patterns [57,58].

Herein, we enrolled a cohort of 104 family trios from Oman, each consisting of an ASD-affected individual and both unaffected parents, and we utilized GS to identify de novo and inherited genetic variants that might contribute to disease risk. Using rigorous selection criteria, we identified 83 ASD candidate genes, including 35 genes previously associated with NDDs in global populations, as well as 48 genes not associated with ASD previously. This study provides a comprehensive genetic investigation of ASD in the Omani population, laying a critical foundation for understanding the genetic complexity of ASD in a region with high consanguinity.

## 2. Results

### 2.1. Cohort Description

The current study was approved by the Medical Research Ethics Committee of Sultan Qaboos University in Muscat and the Institutional Review Board of Qatar Biomedical Research Institute. Written informed consents were obtained from the parents, following strict adherence to the ethical tenets of the Declaration of Helsinki. A total of 312 participants, comprised of 104 probands and both parents, were recruited primarily from the clinics in the Genetics and Developmental Medicine departments at Sultan Qaboos University hospitals. Recruitment of the cohort and the sample collection took place between the years of 2015 and 2022 in the Muscat and Batinah governorates, consisting of densely populated Omani populations. All participants were Omani nationals, and all the parents were negative for autistic traits. The cohort consists of 69 males and 35 females. A total of 59 (57%) families self-reported consanguinity in their marital history, with 61% male and 39% female probands among consanguineous families. Each subject received a clinical diagnosis of ASD based on the criteria outlined in the DSM-5 by the American Psychiatric Association (2013) [4]. Potential confounding variables, including environmental exposures, birth complications, and other pregnancy-related conditions, were evaluated for possible exclusion from the study throughout the recruitment phase. Within this cohort, the most frequently observed comorbidities were language and speech delays (78%), behavioural problems (hyperactive, aggressive, and self-injurious behaviours) (60%), developmental delays (42%), intellectual disability (13%), and epilepsy (12%) (Table 1).

### 2.2. Identification of Candidate ASD Risk Genes in the Omani ASD Cohort

We performed genome sequencing (GS) on both parents and their ASD-affected children (“trios”) from each of the recruited 104 families. The GS data were aligned to the human genome reference sequence (hg38 version), and raw Fastaq files were converted to variant call format (VCF) files using the DRAGEN genomics pipeline v.4.2.4 [59]. Two types of VCF files were obtained, one for single nucleotide variants (SNVs) and another for structural and copy number variants (CNVs). For SNVs, VCF files were analysed using QCI Interpret by Qiagen (https://apps.ingenuity.com/qci) (accessed on 10 March 2024) to identify and prioritize genomic variants associated with ASD. The final GS dataset analysis workflow began with VCF, followed by filtering variants based on multiple criteria, including sequence quality, predicted deleteriousness of the encoded protein, and presence of the variant in common databases such as gnomAD, ExaC (>0.1%). Key filtering criteria to prioritize variants with a predicted deleterious effect on protein function included a high (>20) Combined Annotation Dependent Deletion (CADD) score. The pathogenicity of the identified variants was assessed using guidelines from the American College of Medical Genetics and Genomics (ACMG), along with gnomAD frequency, CADD scores, and various other bioinformatic predictions. We focused on variants classified by ACMG as “variants of unknown significance” (VUS), “likely pathogenic”, or “pathogenic”. Variants classified as “benign” or “likely benign” were excluded from further analysis.

We hypothesized that the probands would carry either known or novel risk gene variants, arising de novo or inherited in autosomal dominant heterozygous, autosomal recessive homozygous, or X-linked recessive hemizygous patterns. Several bioinformatics software packages were used to assess the conservation of these variants and estimate their potential negative impact on gene function. Variants with a minor allele frequency of less than 0.1% that resulted in distinct protein-altering changes (nonsense, insertions or deletions, missense, and splice sites) were prioritized using various protein prediction software (SIFT, PolyPhen-2, CADD, and M-CAP).

### 2.3. Refinement and Prioritization of ASD Candidate Genes

Initially, we obtained 126 variants across 116 different genes from the analysis of 104 trio datasets (Table 2). To further refine these findings, we incorporated gene loss tolerance metrics using calculated Z-scores and pLI scores (indicating the likelihood of being intolerant to loss-of-function). For de novo variants, particularly nonsense/frameshift variants, we applied a strict pLI score threshold greater than 0.9; missense variants were prioritized using a dual criterion: a Z-score above 3.0 combined with a pLI score greater than 0.9. Variants in ASD candidate genes that did not meet these criteria were excluded from further consideration. pLI and Z-scores are primarily developed for evaluating heterozygous variants and the impact of haploinsufficiency. Genes associated with dominant disorders tend to have higher pLI scores because even one functional copy of the gene being disrupted can cause a disorder. In contrast, recessive genes can tolerate LoF mutations in one allele because the other functional copy compensates, leading to lower pLI scores. Homozygous variants in genes with low pLI scores are often mistakenly considered benign, yet they can cause severe disease in recessive disorders. Our final analysis revealed 90 SNVs from 83 unique candidate genes (Table 2).

**Figure 1:**
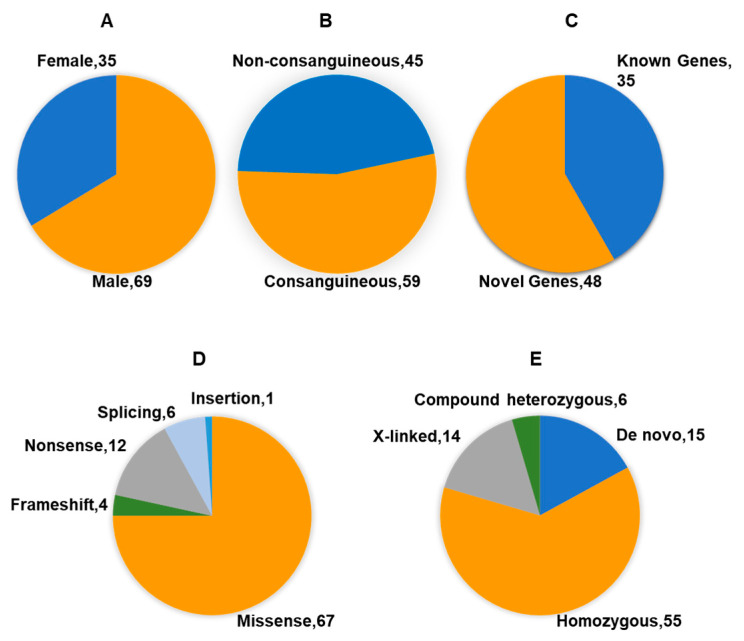
Summary of 104 ASD individuals and their GS results. (**A**) Sex distribution of participants; (**B**) number of consanguineous families; (**C**) counts of known and novel genes identified; (**D**) types of genetic variants identified; (**E**) inheritance patterns of the variants.

The biological and clinical relevance of these genes and their pathways was evaluated through a literature review and by considering the participants’ clinical features. The identified variants included 1 insertion, 4 frameshift, 6 splicing, 12 nonsense, and 67 missense changes. Based on the inheritance pattern, we identified 15 de novo, 55 homozygous, 6 compound heterozygous, and 14 X-linked variants distributed across 35 female and 69 male probands (Table 2 and Figure 1). Detailed criteria and supporting evidence for each variant are provided in Table 2.

### 2.4. Identification of Copy Number Variants (CNV) in the Omani ASD Cohort

As previously described, the original GS data in FASTAQ files were converted to VCF files using the DRAGEN genomics pipeline. CNV detection was then performed using DRAGEN CNV workflow, which employs the GATK Germline CNV caller, a read-depth and junction-based workflow for identifying CNVs > 50 bp [59,60]. We focused exclusively on de novo CNVs. We confidently identified de novo CNVs in eight probands from families 19, 25, 52, 54, 74, 75, 89, and 98, exhibiting heterozygous microdeletions or microduplications ranging from 1 kb to 68 kb (Table 3). Subject 19 displayed an 1173 bp deletion spanning intron-3 to the 5′-UTR of *RBFOX1*, a well-known ASD gene [61,62,63,64]. Subjects 25 and 52 had deletions involving *FGF12* and *SEMA6B*, two known genes associated with ASD and epilepsy [65,66,67,68]. However, these two cases did not report epilepsy. Subjects 54, 74, 89, and 98 had CNVs in other previously reported neurodevelopmental genes, including *BFSP2*, *PCDH15*, *TRPM3*, *TPO*, *PDSS1*, and *SLCO1B1* [69,70,71,72,73,74]. Collectively, ten de novo CNVs identified in eight ASD individuals are either intragenic or affect one end of a gene, involving a total of ten candidate genes. Notably, several sporadic variants in these genes have been documented in patients with ASD, intellectual disability (ID), development disorders, or other NDDs in the literature, further supporting their role as ASD candidate genes [69,70,71,72,73,74] (Table 3).

### 2.5. Identification of 35 Known NDD-Associated Genes

To further validate our results, we compared our newly identified gene list of 83 genes with the established neurodevelopmental disorder (NDD)/ASD gene databases, including the Genomics England NDD/Autism panel (https://panelapp.genomicsengland.co.uk/panels/285) (accessed on 1 August 2024), the Simons Foundation Autism Research Initiative (SFARI) gene list (https://gene.sfari.org/database/human-gene) (accessed on 1 August 2024), and The Human Gene Mutation Database (HGMD) (https://apps.ingenuity.com) (accessed on 1 August 2024). Genes from our list that were also present in these established databases were marked as present (“Known”) or not present “Novel” in Table 2. This comparison revealed that only 3 out of the 83 genes overlapped with the SFARI genes (*LAS1L*, *PHF14*, and *SLC6A1*), and only 7 genes matched the Genomics England NDD/Autism panel genes (*HTT*, *LAS1L*, *MYO7A*, *PHF14*, *PRRT2*, *SLC6A1*, *XYLT1*). In a reverse approach, 35 of our genes, which showed mutations across multiple subjects, were found in individuals with various NDDs such as ASD, ID, macrocephaly, and other developmental disorders, as well as neurodegenerative disorders, including Huntington’s disease, Parkinson’s disease, and ALS according to the HGMD database (Table 2). This overlap corroborates the relevance of these genes in ASD, suggesting they may play a key role in the disorder since they are already known to cause various neurodevelopmental and/or neurodegenerative disorders (Table 2).

### 2.6. Identification of 48 Novel ASD-Risk Genes

Many genes associated with NDDs may also play a role in ASD [75,76]. In this study, we employed “parent-offspring trio genome sequencing” to analyse 104 subjects with ASD, identifying 83 genes with a strong likelihood of contributing to ASD. We followed ACMG guidelines and applied rigorous criteria, including low population prevalence; high-quality sequencing coverage; elevated CADD scores, pLI, and Z-scores to prioritize and finalize the candidate gene list. As mentioned above, 35 of these genes were previously associated with NDDs or ASD, which we designate as “Known” (Table 2). In addition, we also identified 48 candidate genes not previously associated with ASD, which we refer to hereafter as “Novel” candidate genes. These novel candidate genes have limited or no information regarding their involvement in ASD, and their specific roles in ASD phenotype have not yet been described in the literature (Table 2). These novel genes contribute to a variety of biological processes, such as protein–protein and protein–DNA interactions, chromatin or DNA binding, and GTPase-activating functions, highlighting the complex genetic basis of ASD.

### 2.7. Sporadic Variants Identified in Our Novel ASD Candidate Genes

Advances in NGS have generated vast genome data from various diseases, including diabetes, cancer, cardiovascular disorders, neurodegenerative disorders, and NDDs such as ASD [32,38,45,77,78,79,80,81,82,83,84,85,86,87,88]. These genomic data provide a unique opportunity to characterize the biological functions of genes and elucidate the molecular mechanisms underlying these developmental disorders. HGMD is a comprehensive repository that catalogues genetic variants implicated in human disorders. It includes information on mutations with confirmed roles in disease causation, as well as sporadic variants found in potential disease genes reported in the literature [89]. We combed through the HGMD against our list of newly identified novel ASD candidate genes and found that most of these genes had genomic variants, in the form of SNVs or CNVs, previously linked to NDDs and neuro-degenerative disorders [29,30,31,38]. This raises the possibility that these genes could potentially serve as ASD candidate genes, even though they have not been formally classified as ASD-linked or ASD-susceptibility genes and are not included in SFARI or NDD/ID gene panels. Given the sporadic variants reported for these genes in HGMD, it is likely that they could be involved in disease causation.

### 2.8. Protein–Protein Enrichment Analysis of Candidate Genes

**Figure 2:**
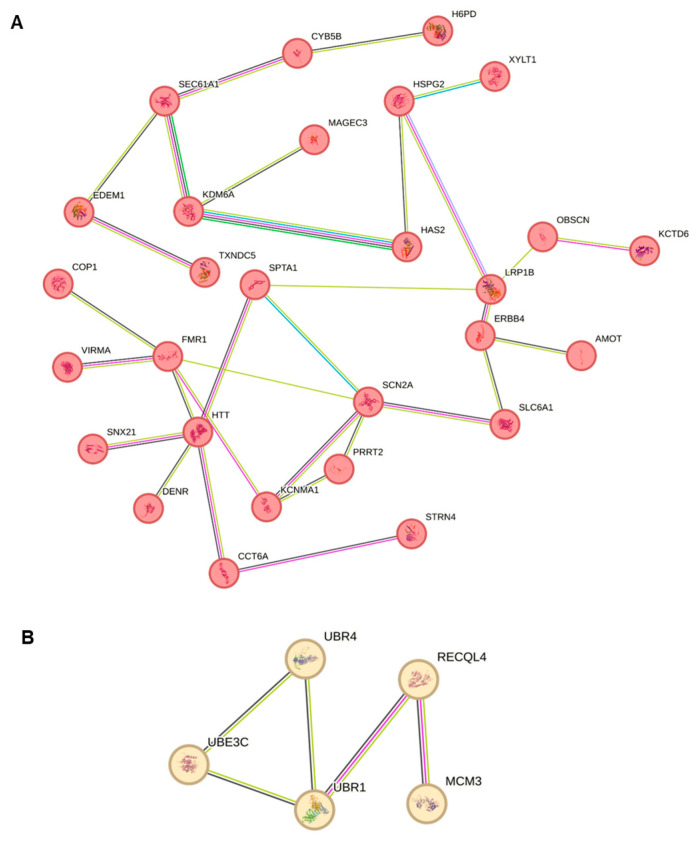
STRING interaction enrichment analysis identified two dominant enrichment networks as denoted by red (synaptic membranes—(**A**)) and yellow nodes (ubiquitin pathway—(**B**)). Coloured nodes represent query proteins and the first shell of interactions, connected by coloured lines indicating the known interactions derived from experimental data, curated databases, co-expression studies, or text-mining. Blue lines denote known interactions from curated databases, pink lines represent experimentally determined interactions, black lines indicate co-expression, and purple lines signify homology.

We performed protein–protein interaction enrichment analysis using STRING 3.0 (https://string-db.org/) (accessed on 1 September 2024) on our candidate genes (Supplementary Figure S1). Upon k-means clustering, this analysis revealed two predominant networks among the candidate genes identified (Figure 2A,B). The first network consisted of 28 nodes and 31 edges (average node degree: 2.21, average local clustering coefficient: 0.463, and PPI enrichment *p* value: 5.23 × 10^−10^) consisting of 28 proteins associated with components of the presynaptic and postsynaptic membranes. These genes include *AMOT*, *CCT6A*, *COP1*, *CYB5B*, *DENR*, *EDEM1*, *ERBB4*, *FMR1*, *H6PD*, *HAS2*, *HSPG2*, *HTT*, *KCNMA1*, *KCTD6*, *KDM6A*, *LRP1B*, *MAGEC3*, *OBSCN*, *PRRT2*, *SCN2A*, *SEC61A1*, *SLC6A1*, *SNX21*, *SPTA1*, *STRN4*, *TXNDC5*, *VIRMA*, and *XYLT1* (Figure 2A). The presynaptic membrane is essential for neurotransmitter release and synaptic transmission, processes fundamental to brain function. In ASD, abnormalities in presynaptic mechanisms have been implicated as contributors to the neurobiological features [90,91,92]. The second network consisted of 5 nodes and 5 edges (average node degree: 2, average local clustering coefficient: 0.667, and PPI enrichment *p* value: 2.71 × 10^−6^) and was characterized by ubiquitin-conjugating enzyme activities involving genes such as *UBR1*, *UBR4*, *UBE3C*, *RECQL4*, and *MCM3* (Figure 2B). Ubiquitin-conjugating enzymes play a vital role in the ubiquitin–proteasome system, which regulates protein degradation and turnover, thereby maintaining cellular homeostasis and various cellular functions. With respect to ASD, disruptions in ubiquitin-conjugating enzyme activity could affect protein homeostasis, synaptic function, inflammation, stress responses, as well as neuronal differentiation, migration, and synaptogenesis, which are all relevant to neurodevelopmental processes [93,94,95].

## 3. Discussion

This study employed GS to identify genetic variants associated with ASD in a cohort from Oman, addressing the underrepresentation of genetic studies on ASD in the Middle East. The discovery of ASD genes has been hampered by high genetic heterogeneity, with many genes each contributing to small, complex effects that require large sample sizes to detect. ASD is also linked to rare variants in the general population and de novo variants in affected individuals, which are challenging to detect without diverse populations and advanced analytical approaches. Especially in Arab populations, genetic studies on ASD remain fragmentary compared to the extensive research conducted in Western populations, limiting insights into ASD’s genetic landscape in this region. To address this gap, we performed GS on 104 unrelated families affected by ASD of unknown etiology in Oman, employing a trio analysis, which sequences the genomes of an affected individual and both unaffected biological parents. This approach offers several advantages over singleton sequencing, particularly in identifying de novo and inherited mutations. By examining inheritance patterns, we can classify variants as benign or pathogenic more effectively, which aids in pinpointing those most likely associated with ASD. Comparing the proband’s genome with that of both parents also helps filter out common variants, thereby reducing the number of irrelevant findings. Finally, for families with suspected rare syndromic ASD, trio sequencing significantly improves diagnostic yield by uncovering rare or novel variants that singleton sequencing might miss.

Our research identified 116 unique genes across 95 families, while 10 CNVs were detected in 8 families. Of the 116 genes identified, 83 are considered strong candidate genes for ASD based on ACMG guidelines, comprising 15 de novo, 55 homozygous, 6 compound heterozygous, and 14 hemizygous variants. Among these 83 genes, 48 are novel candidate genes significantly associated with ASD in the Omani cohort, while the remaining 35 are known ASD genes. Ten de novo micro-CNVs identified in eight ASD subjects are either intragenic or affect one end of a gene, involving a total of ten candidate genes (Table 3). Notably, at least several sporadic variants in these genes have been reported in patients with ASD, intellectual disability (ID), developmental disorders, or other NDDs in the literature, further supporting their potential role as ASD candidate genes.

STRING protein–protein interaction enrichment analysis of these genes provided molecular etiological insights, demonstrating connections between ASD and fundamental cellular functions such as presynaptic membrane signaling and ubiquitin-conjugating enzyme pathways.

Most of the variants identified in our limited cohort were either absent in the gnomAD and other polymorphism databases or present at very low frequencies (<0.01%). Furthermore, these variants displayed very low frequencies in the Qatar Genome Program (QGP) database, which includes genomic data from over 10,000 healthy individuals from Qatar [21]. The strength of our methodology was further validated by identifying variants in NDD genes or ASD genes, as documented in high-throughput sequencing databases and the literature.

An important feature of our Omani cohort was that, out of 104 families, 56 self-reported consanguineous marriages. Among these 56 families, we identified 53 strong candidate genes across 41 families. Of these 53, 47 variants were inherited from parents as either homozygous recessive (41 genes) or X-linked (6 genes). Interestingly, we also identified de novo variants in 6 families, indicating that consanguineous families can harbor both de novo and recessive variants [96]. A higher prevalence of recessive mutations suggests that consanguinity plays an important role in the genetic etiology of ASD in this region.

One aim of this study was to identify variants specific to the Arab/Oman population. Interestingly, we found seven genes with putative causative variants recurring across families: six genes appeared in two families each, and one gene *DNAH17* (dynein axonemal heavy chain 17, MIM 610063) was identified in three different families (family numbers 31, 66, and 84) with compound heterozygous variants. The nucleotide variants were identical in families 31 and 84 (c.2121C>A and c.12650T>C) but differed in family 66 (c.12389C>T and c.11825G>A). Loss of function mutations in *DNAH17* are linked to a genetically heterogeneous disorder leading to male infertility and multiple morphological abnormalities of the flagella (MMAF) [97]. However, mutations in the *DNAH17* potentially affect neuronal function, synaptic connectivity, or ciliary-related signaling pathways important for brain development [98,99]. This is supported by sporadic missense and nonsense variants in ASD patients documented in the HGMD [30,31,100].

An *FMR1* variant c.716C>T was identified in two families (22 and 91). While CGG repeat expansions in *FMR1* are known to cause ID, point mutations in *FMR1* have also been reported in ID subjects [101,102]. The *FMR1* gene (MIM 309550), located on the X-chromosome, is one of the most extensively studied genes in relation to ASD, primarily due to its role in Fragile X Syndrome (FXS) (MIM 300624) [103,104]. *FMR1* encodes the Fragile X Mental Retardation Protein (FMRP), a key regulator of synaptic plasticity and local protein synthesis at synapses [104]. In individuals with FXS, the absence or deficiency of FMRP impairs synaptic function, leading to ID, ASD, and other NDDs [105].

*SCN2A* (MIM 182390) encodes the alpha subunit of the Nav1.2 voltage-gated sodium channel, which is critical for neuronal function, particularly in initiating and propagating action potentials. Variants in *SCN2A* have been strongly associated with NDDs, including ASD [106,107,108,109]. In our study, two different de novo *SCN2A* variants were identified: c.3932T>G in family 49 and c.532G>T in family 90.

*GIPR* (MIM 137241) encodes the receptor for gastric inhibitory polypeptide, a hormone primarily involved in regulating insulin secretion and lipid metabolism [110,111]. *GIPR* plays a critical role in metabolic regulation, the gut–brain axis, and potential neuroinflammatory mechanisms [110,111,112]. Although *GIPR’s* primary functions are linked to metabolic pathways, disruptions in these processes may affect neurodevelopment and brain function, potentially contributing to ASD pathophysiology. We identified two *GIPR* variants: c.1152G>T and c.302G>A in family 96 and c.1264C>T in family 100.

The same variant c.1879C>T in *SPATA31A3* was identified in family 44 and family 58. *SPATA31A3*, a member of the SPATA (Spermatogenesis-Associated) gene family, is involved in multiple cellular processes, including DNA repair and the maintenance of genomic stability, both of which are crucial for normal brain development and function [113,114]. Two sporadic missense variants in this gene have been reported in ASD patients [30].

We identified an identical *TRIM15* variant (c.608A>G) in families 18 and 80 and 1 same variant in *TRIM73* (c.487C>T) in families 3 and 77. *TRIM15* and *TRIM73* (MIM 612549) are members of the TRIM (Tripartite Motif-containing) protein family, which participates in various biological processes, including immune response, cell proliferation, and gene expression regulation [115,116,117,118]. While the exact roles of *TRIM15* and *TRIM73* in ASD are not fully understood, emerging evidence suggests their potential involvement in neurodevelopment and immune regulation, both of which are relevant to ASD pathology [115,116,117,118]. A regulatory variant in *TRIM15* and a missense variant in *TRIM73* have been reported in ASD patients [30,31].

Identifying multiple variants of the same gene in independent patients strengthens the evidence for the gene’s potential role in disease etiology. This recurrence suggests that the gene may be functionally important to the condition of ASD and helps distinguish disease genes from incidental findings. Recurrent variants in the same gene across different patients also guide further functional studies, improve genetic diagnosis, and may support the development of targeted therapeutic approaches. Furthermore, the identification of multiple genes linked to ASD within a specific population provides insights into the unique genetic architecture and risk factors of ASD in that population. Such findings can reveal population-specific variants and gene–environment interactions that may be absent or less prevalent in other groups. This knowledge improves the accuracy of genetic diagnoses and risk assessments within that population, facilitates tailored genetic counselling, and contributes to the development of population-specific treatments or interventions. Additionally, understanding these genetic variations enhances global ASD research by adding diversity to genetic databases, which is essential for identifying both shared and unique mechanisms underlying ASD across populations.

## 4. Materials and Methods

### 4.1. Sampling Procedures and DNA Isolation

Bio-samples collected for this study mainly consisted of blood from affected individuals and their parents. Samples were collected at Sultan Qaboos University, Muscat, Oman, in tubes treated with anti-coagulant ethylenediamine tetra acetic acid (EDTA) to preserve blood for DNA and RNA extraction. Genomic DNA was extracted from peripheral blood leukocytes using the Flexigene DNA extraction kit protocols (Qiagen, Hilden, Germany). DNA concentrations were measured using a Nanodrop Spectrophotometer 1000 (ND-1000; Thermo Fisher Scientific, Waltham, MA, USA) and further validated for quality using a Qubit fluorometer (ThermoFisher Scientific, Waltham, MA, USA). Finally, DNA samples were diluted and aliquoted to 2 μg per sample in new barcoded vials, which were labeled and prepared for shipment to Qatar Genome Program in Qatar for genome sequencing.

### 4.2. Library Construction and Genome Sequencing

GS for this study adhered to established protocols, incorporating rigorous quality control to ensure accuracy, reliability, and reproducibility. These measures reduce errors and prevent sample contamination to generate high-quality data [21]. Library construction and sequencing were conducted at the Sidra Clinical Genomics Laboratory Sequencing Facility, utilizing the Agilent SureSelectXT kit from Agilent Technologies, Santa Clara, CA, USA, following the manufacturer’s instructions. The workflow began with mechanical fragmentation of 200 ng of genomic DNA using the Covaris E220 ultrasonicator system, Covaris PerkinElmer, Waltham, MA, USA followed by DNA purification with AMPure XP magnetic beads, Beckman Coulter, Indianapolis, IN, USA. The fragmented DNA was subsequently end-repaired, adenylated, and ligated to SureSelect DNA adapters, Agilent Technologies, Santa Clara, CA, USA. After ligation, the DNA was further purified and amplified by PCR. The amplified library was then hybridized to biotin- labeled probes targeting specific regions of interest, captured with streptavidin-coated beads, Agilent Technologies, Santa Clara, CA, USA and subjected to a second round of PCR with a unique index. Library quality was assessed using the Agilent 2100 Bioanalyzer, and concentrations were quantified with the Qubit system, ThermoFisher, Waltham, MA, USA. Libraries meeting quality control criteria were pooled and sequenced on the Illumina HiSeq 4000 platform, generating a minimum of 50 million paired-end reads (2 × 150 bp) per sample.

### 4.3. Variant Calling Process

Sidra Research, Clinical Genomics Laboratory (CGL) in Qatar provided GS data in Fastq format. This raw FastaQ data were further processed using Illumina’s DRAGEN (Dynamic Read Analysis for GENomics) platform,(v.4.2.4) a high-performance solution optimized for GS data analysis [59,60]. DRAGEN pipeline starts by ingesting raw sequencing data in Fastq format by applying initial quality checks to assess read quality metrics such as base quality scores, GC content, and adapter content. Subsequently, the reads are aligned to reference genome (GRCh38) using DRAGEN’s hardware-accelerated mapping and alignment algorithm, known as “hash table alignment”, which enables efficient and rapid reference-based mapping. DRAGEN then identifies and marks duplicate reads, and base quality scores are recalibrated to correct for systematic biases observed during sequencing process by analysing empirical error rates across various sequence contexts. Variant calling for SNVs and insertions/deletions (indels) is conducted using probabilistic models adapted from GATK’s HaplotypeCaller, optimized for DRAGEN’s architecture. Beyond SNVs and indels detection, DRAGEN also identifies structural variants (SVs) in chromosomes, such as CNVs, large deletions or duplications, and translocations. Haplotype phasing is performed to assign variants to maternal or paternal chromosomes, enhancing the interpretability of variant calls and inheritance patterns, particularly in compound heterozygous variants. Finally, identified variants are annotated using databases such as ClinVar, dbSNP, and other population and clinical variant resources and presented in annotated VCF files for downstream analysis [59,60]

### 4.4. Integrating Genetic Databases to Validate ASD Candidate Genes

To validate the significance of our ASD candidate genes, we performed gene annotation using three comprehensive resources: the SFARI gene database https://gene.sfari.org/database/human-gene/ (accessed on 1 August 2024), the Genomics England NDD/Autism gene panel https://panelapp.genomicsengland.co.uk/panels/285/ (accessed on 1 August 2024), and HGMD https://my.qiagendigitalinsights.com/bbp/view/hgmd/pro/all.php (accessed on 1 August 2024). The SFARI database catalogs genes potentially implicated in ASD, while the Genomics England NDD/Autism panel includes genes associated with various NDDs, including autism, epilepsy, ID, attention deficit hyperactivity disorder (ADHD), and other NDDs. HGMD, on the other hand, includes both causative variants linked to known human disorders and sporadic variants identified in patients with specific disorders, even if their causality has not been definitively established. This resource offers a comprehensive collection of genetic variants reported in the literature, encompassing both pathogenic mutations and variants of uncertain significance associated with inherited diseases [89]. We cross-referenced our newly identified gene list with the following resources: SFARI, Genomics England NDD/ASD, and HGMD. Moreover, we categorized each gene as “Known” or “Novel” in Table 2 based on their presence in these databases. This comparison aligns our results with well-established databases, enhancing the relevance of our findings and supporting the potential involvement of these genes in ASD.

### 4.5. STRING Interaction Enrichment Analysis

The STRING 3.0 analysis was utilized for our candidate genes to explore and validate protein–protein interaction (PPI) within this unique gene set. By mapping these genes to STRING, we aimed to identify functional networks and interaction clusters that could reveal biological pathways and mechanisms potentially underlying ASD in Omani population. This approach enabled us to integrate multiple interaction sources, including text mining, experimental data, curated databases, and co-expression while restricting our analysis to “homo sapiens” and applying an interaction score threshold of >0.4 to construct the PPI networks. Finally, functional clusters within these PPI networks were subsequently identified using k-means clustering. Each node represents a protein with its 3D structure displayed, while edges signify protein–protein interactions. Blue lines denote known interactions from curated databases, pink lines represent experimentally determined interactions, black lines indicate co-expression, and purple lines signify homology. A low PPI enrichment *p*-value suggests a non-random network, indicating that the observed number of edges is statistically significant.

## 5. Conclusions

This study identified ASD candidate genes in an Omani cohort, shedding light on the genetic underpinnings of ASD in this population. We have identified 48 novel ASD candidates and 35 genes previously linked to NDDs. The presence of multiple candidate variants in some individuals suggests potential digenic or polygenic inheritance. The high prevalence of homozygous variants, 61% in the entire cohort and 84% in consanguineous families supports a recessive genetic architecture for many ASD cases in this population, consistent with the high rates of consanguinity.

These findings underscore the importance of considering population-specific genetic factors and suggest that recessive alleles contribute significantly to ASD risk in this region. This work also highlights the value of examining rare, potentially pathogenic variants in consanguineous populations, which may improve genetic counseling and inform public health initiatives, including genetic screening in Oman and the Middle East. Identifying new ASD-associated genes provides insights into the disorder’s genetic mechanisms and could guide the development of diagnostic tools, such as gene panels, for early and precise diagnosis. Ultimately, these discoveries open avenues for personalized treatment strategies, potentially enabling more targeted and effective interventions for ASD. Further research on these genetic pathways will be essential for developing therapies that improve outcomes for individuals with ASD.

## Figures and Tables

**Table 1:** Summary of clinical features of Omani cohort with associated comorbidities.

	Number of Individuals (%)
Sex	Male—66%
	Female—34%
Consanguinity (parents)	56%
Ethnicity	100%
Autism	100%
Language/speech delay	78%
Behavioural problems	60%
Developmental Delays	42%
Intellectual disability	13%
Epilepsy/seizures/spasms	12%

All the parents were negative for autistic traits.

**Table 2:** Overview of single nucleotide variants identified from genome sequencing (GS) analysis of 104 Omani autism individuals.

Subject	Gender	Consanguinity	Genes	Accession Number	Chr.	Nucleotide Change	Protein Change	Type of Variant	Inheritance (GENOTYPE)	gnomAD (AF)	QGP (AF)	CADD	ACMG Interpretation	Z Score	pLI Score	Previously Linked with ASD/NDDs
1	M	+	*HNRNPCL4*	NM_001302551.2	1	c.830C>T	p.A277V	missense	homoz.	0	0	21.2	LP	NA	NA	Novel
2	M	-	*PNCK*	NM_001366977.1	X	c.932G>A	p.R311Q	missense	x-linked	0	0.000238598	21.1	LP	NA	NA	Novel
3	M	-	*COP1*	NM_022457.7	1	c.1291C>T	p.R431W	missense	de novo	0	0	25.8	P	−0.36	0.92	Novel
			*TRIM73*	NM_198924.4	7	c.487C>T	p.R163*	nonsense	homoz.	0	0	35	P	3.23	0.93	Novel
4	M	+	*TSKU*	NM_015516.4	11	c.979C>T	p.R327W	missense	homoz.	0	0	25	LP	1.22	0.17	Novel
5	F	-	*SLC6A1*	NM_003042.4	3	c.1328G>T	p.G443V	missense	de novo	0	0	27.9	P	4.92	1	Known
			*CAPN8*	NM_001143962.2	1	c.730-2A>G	splicing	splicing	de novo	0	0	25.9	P	−0.19	0	Novel
			*MYO7A*	NM_000260.4	11	c.2489G>A	p.R830H	missense	homoz.	0.0114, European	0.00214739	32	VUS	0.06	0	Known
6	M	-	*RAB11FIP3*	NM_014700.4	16	c.1396-2A>G	splicing	splicing	de novo	0	0	35	P	−0.86	1	Novel
7	M	+	*XYLT1*	NM_022166.4	16	c.1129C>G	p.Q377E	missense	homoz.	0	0.0144863	22.3	LP	0.75	0.99	Known
8	F	-	*SYTL1*	NM_001193308.2	1	c.1317_1323delAGGATCC	p.G440fs*?	frameshift	de novo	0	0		P	−0.52	0	Novel
9	M	-	*LAS1L*	NM_031206.7	X	c.1082C>G	p.P361R	missense	X-linked	0	0.00572636	22.1	LP	NA	NA	Known
10	M	-	*RIBC1*	NM_001031745.5	X	c.106C>G	p.R36G	missense	X-linked	0	0	23.9	LP	NA	NA	Novel
11	F	+	*KIAA1755*	NM_001029864.2	20	c.2052+1G>A	splicing	splicing	homoz.	0.0024, African	0.00105665	33	P	−0.24	0	Novel
12	M	-	*EDEM1*	NM_014674.3	3	c.1301G>A	p.W434*	nonsense	de novo	0	0	45	P	−1.39	0	Novel
			*SLC12A9*	NM_020246.4	7	c.764A>C	p.Y255S	missense	de novo	0	0	28.5	LP	1.96	0	Novel
13	M	-	*AMOT*	NM_001113490.2	X	c.514C>T	p.R172C	missense	X-linked	0	0	29	LP	NA	NA	Novel
14	F	+	*VWA8*	NM_015058.2	13	c.5093G>A	p.R1698Q	missense	homoz.	0.0038, European	3.40855 × 10^−5^	29.9	VUS	0.51	0	Novel
			*H6PD*	NM_004285.4	1	c.127T>C	p.W43R	missense	homoz.	0.0013, European	3.40855 × 10^−5^	29.6	VUS	0.11	0	Novel
			*KIAA1755*	NM_001029864.2	20	c.19G>A	p.D7N	missense	homoz.	0	0.000340855	25.3	LP	−0.24	0	Novel
15	M	+	*TTC36*	NM_001080441.4	11	c.284G>C	p.R95P	missense	homoz.	0	0	23.6	LP	−1.92	0	Novel
16	M	-	*LILRB3*	NM_006864.4	19	c.1050T>G	p.Y350*	nonsense	homoz.	0	0	23.2	P	1.45	0	Novel
			*TMEM31*	NM_182541.2	X	c.142C>A	p.Q48K	missense	X-linked	0.0094, Latino	0.000443111	21.7	VUS	NA	NA	Novel
17	M	+	*PRRT2*	NM_145239.3	16	c.649dupC	p.R217fs*8	frameshift	de novo	0	0	31	P	−0.26	0.65	Known
18	F	+	*TRIM15*	NM_033229.3	6	c.608A>G	p.D203G	missense	de novo	0	0	23.5	LP	−0.18	0	Novel
			*LTBP1*	NM_206943.4	2	c.3616T>C	p.C1206R	missense	homoz.	0	0	29.8	LP	−0.7	0	Novel
19	M	-	CNV identified													
20	M	+	*ATP13A5*	NM_198505.4	3	c.632A>G	p.Q211R	missense	de novo	0	0	26.1	LP	1.24	0	Novel
			*COL6A6*	NM_001102608.3	3	c.5002G>T	p.G1668*	nonsense	homoz.	0	0.000340855	47	P	0.53	0	Novel
21	M	-	*RORC*	NM_005060.4	1	c.253C>T	p.H85Y	missense	homoz.	0.0654	0.0116572	26.9	VUS	2.89	1	Novel
22	M	+	*ERBB4*	NM_005235.3	2	c.2900T>G	p.F967C	missense	homoz.	0	0.000204513	31	LP	2.82	1	Known
			*FMR1*	NM_002024.6	X	c.716C>T	p.A239V	missense	X-linked	0	0	25.3	LP	NA	NA	Known
23	M	+	*ECH1*	NM_001398.3	19	c.284A>G	p.K95R	missense	homoz.	0	0	26	LP	−0.17	0	Novel
24	M	+	*ZNF250*	NM_001109689.4	8	c.593A>T	p.E198V	missense	de novo	0	0	23.4	LP	3.41	0.93	Novel
			*AGTR2*	NM_000686.5	X	c.411C>A	p.C137*	nonsense	X-linked	0	3.40855 × 10^−5^	31	P	NA	NA	Known
25	F	-	CNV identified													
26	F	_	*DENND2A*	NM_015689.5	7	c.2971G>A	p.G991S	missense	homoz.	0.0024, African	0.000204513	25.8	LP	1.84	0	Novel
27	M	_	*HTT*	NM_001388492.1	4	c.102_110dupGCAGCAGCA	p.Q36_Q38dup	in-frame Insertion	de novo	0	0	NA	LP	3.7	1	Known
28	F	_	*HIVEP3*	NM_024503.5	1	c.3316C>T	p.Q1106*	nonsense	de novo	0	0	35	P	NA	NA	Novel
29	M		*PLIN4*	NM_001367868.2	19	c.2246_2247insG	p.G750fs*14	frameshift	de novo	0.0151, Latino	0	23.9	P	−1.44	0	Novel
			*RAB3B*	NM_002867.4	1	c.8C>T	p.S3L	missense	de novo	0	0	23.7	LP	NA	NA	Novel
			*PGAP3*	NM_033419.5	17	c.717G>C	p.W239C	missense	homoz.	0.0048, African	0	31	VUS	-0.47	0	Novel
30	M	+	*LMF1*	NM_022773.4	16	c.529C>T	p.L177F	missense	homoz.	0	0.00146568	28.2	LP	−2.56	0	Known
31	M	_	*DNAH17*	NM_173628.4	17	c.12650T>C	p.I4217T	missense	comp. het.	0	0	26.9	LP	−2.01	0	Known
					17	c.2121C>A	p.N707K	missense		0	3.40855 × 10^−5^	10	LP			
32	M	_	*AGAP9*	NM_001190810.1	10	c.1930_1931insTCTGGTA	p.T644fs*?	frameshift	homoz.	0	0	NA	P	4.15	0	Novel
33	M	+	*OBSCN*	NM_001386125.1	1	c.2186A>C	p.E729A	missense	homoz.	0	0.00654441	25	LP	−0.82	0	Known
			*HAS2*	NM_005328.3	8	c.221C>G	p.S74C	missense	homoz.	0	0	23.4	LP	4.42	1	Novel
			*ZNF81*	NM_007137.5	X	c.980A>G	p.E327G	missense	X-linked	0.0188, Latino	0.000579493	24.2	VUS	NA	NA	Known
34	M	-	*MAP3K9*	NM_001284230.2	14	c.1790G>A	p.R597Q	missense	de novo	0	3.40855 × 10^−5^	23.8	LP	1.6	0	Novel
			*MAGEC3*	NM_138702.1	X	c.212C>A	p.S71*	nonsense	X-linked	0.0017 European	0	26.3	P	NA	NA	Novel
35	F	-	*ABCA13*	NM_152701.5	7	c.633-2A>G	splicing	splicing	de novo	0	0	33	P	−0.12	0	Known
			*CLEC18C*	NM_173619.4	16	c.994G>A	p.G332R	missense	homoz.	0	0	29	LP	3.26	0.94	Novel
36	M	+	*ZNF483*	NM_133464.5	9	c.2047C>T	p.R683*	nonsense	*homoz.*	0	0	35	P	3	0.97	Novel
			*PREP*	NM_002726.5	6	c.1213+1G>A	splicing	splicing	*homoz.*	0.0013 European	3.40855 × 10^−5^	34	P	2.65	1	Novel
37	M	+	*ZNF207*	NM_001098507.2	17	c.637G>C	p.G213R	missense	*homoz.*	0	0	25	LP	2.69	0.12	Novel
			*CARMIL1*	NM_017640.6	6	c.3149A>T	p.H1050L	missense	*homoz.*	0	0.00132933	20.7	LP	2.03	0	Novel
38	F	NA	*KCNMA1*	NM_001161352.2	10	c.2725C>T	p.R909W	missense	de novo	0	0	32	LP	6.52	1	Known
			*TMEM184A*	NM_001097620.2	7	c.365C>T	p.S122F	missense	de novo	0	0	22.8	LP	−0.75	0	Novel
39	M	-	*CRYBG1*	NM_001371242.2	6	c.4916A>T	p.K1639I	missense	*homoz.*	0	0	22.5	LP	0.93	0	Novel
		-	*NUS1*	NM_138459.5	6	c.684T>A	p.S228R	missense	*homoz.*	0	0	22	LP	1.03	1	Known
40	M	+	*KCTD6*	NM_001128214.2	3	c.538G>A	p.G180R	missense	homoz.	0	0	23.1	LP	2.86	0.38	Novel
41	F	+	*PDE2A*	NM_002599.5	11	c.229C>T	p.R77*	nonsense	homoz.	0	0	34	P	3.86	0.02	Known
42	M	_	*ARHGAP33*	NM_001366178.1	19	c.3691C>T	p.P1231S	missense	homoz.	0	0.000272684	21.4	LP	1.78	0	Novel
43	F	+	*UBE2Q2*	NM_173469.4	15	c.1031A>T	p.N344I	missense	homoz.	0.0065	0.000715795	25	VUS	0.38	0	Novel
44	M	+	*SPATA31A3*	NM_001083124.1	9	c.1879C>T	p.Q627*	nonsense	homoz.	0	0	25.9	P	5.51	0.65	Novel
45	M	+	*SORCS2*	NM_020777.3	4	c.1662G>A	p.W554*	nonsense	homoz.	0	0	47	P	−1.75	0	Novel
46	M	+	*CWF19L2*	NM_152434.3	11	c.270G>T	p.K90N	missense	de novo	0	3.40994 × 10^−5^	23	LP	−0.65	0	Novel
47	F	+	*CYB5B*	NM_030579.3	16	c.212G>A	p.G71D	missense	homoz.	0	0	28.5	LP	0.81	0.01	Novel
			*HSPG2*	NM_005529.7	1	c.1369A>G	p.S457G	missense	homoz.	0	0	27.9	LP	3.4	0	Known
48	M	+	*SPTA1*	NM_003126.4	1	c.2195T>A	p.L732Q	missense	de novo	0	0	25.4	LP	−0.8	0	Known
49	F	-	*SCN2A*	NM_001040142.2	2	c.3932T>G	p.L1311R	missense	de novo	0	0	29.3	LP	8.71	1	Known
50	F	+	*NOTCH4*	NM_004557.4	6	c.1522C>T	p.Q508*	nonsense	de novo	0	0	38	P	2.17	0	Novel
			*MTUS1*	NM_001363059.2	8	c.1165C>T	p.Q389*	nonsense	de novo	0	0.00149976	36	P	−5.75	0	Novel
51	M	+	*NHLRC1*	NM_198586.3	6	c.946T>A	p.F316I	missense	de novo	0	0	22.4	LP	−0.09	0	Known
52	F	-	CNV identified													
53	M	+	*SEC61A1*	NM_013336.4	3	c.331C>G	p.L111V	missense	homoz.	0	0	23.4	LP	4.94	1	Novel
54	F	+	CNV identified													
55	M	+	*RNF113A*	NM_006978.3	X	c.76G>A	p.G26R	missense	X-linked	0	0.00037494	21.7	LP	NA	NA	Novel
56	M	-	*TNRC18*	NM_001080495.3	7	c.487+126delT	Intronic	intronic	de novo	0	0	NA	LP	−7.54	0	Novel
57	M	-	*PHF14*	NM_001007157.2	7	c.73A>C	p.S25R	missense	de novo	0	0	29.9	LP	0.87	0.03	Known
58	M	-	*SPATA31A3*	NM_001083124.1	9	c.1879C>T	p.Q627*	nonsense	homoz.	0	0	25.9	LP	5.51	0.65	Novel
59	M	+	*CCT6A*	NM_001762.4	7	c.336+1G>A	Splicing	splicing	de novo	0	3.40855 × 10^−5^	34	P	1.42	1	Novel
60	F	-	*ESYT3*	NM_031913.5	3	c.2468+1G>T	Splicing	splicing	homoz.	0	0.00528325	34	P	0.17	0	Novel
61	M	+	*UBE3C*	NM_014671.3	7	c.916_917delAG	p.S306*	frameshift	homoz.	0	0	NA	P	3.04	1	Novel
62	F	+	*MANSC1*	NM_018050.4	12	c.250T>G	p.F84V	missense	comp. het.	0	0	27.3	LP	0.82	0	Novel
					12	c.38T>A	p.L13*	nonsense			0.00037494	34	P	0.82	0	
63	F	+	*SLC19A1*	NM_194255.4	21	c.1514A>G	p.E505G	missense	homoz.	0	0.00109074	22.9	LP	−0.71	0	Novel
64	M	-	*LRP1B*	NM_018557.3	2	c.1560G>T	p.K520N	missense	homoz.	0	0	26.3	LP	3.33	1	Known
65	M	-	*VIRMA*	NM_015496.5	8	c.1069A>T	p.T357S	missense	de novo	0	3.40855 × 10^−5^	25.2	LP	3.68	1	Novel
			*GRAMD1B*	NM_001387025.1	11	c.973T>C	p.F325L	missense	de novo	0	0	24.2	LP	1.13	0.01	Novel
66	M	+	*DNAH17*	NM_173628.4	17	c.12389C>T	p.P4130L	missense	comp. het	0	0.000102256	27.5	LP	−2.01	0	Known
					17	c.11825G>A	p.R3942Q	missense		1.4	0.00453337	23.5	LP	−2.01	0	
67	M	+	*RNF175*	NM_173662.4	4	c.247-1G>A	splicing	splicing	homoz.	0	3.40855 × 10^−5^	33	P	0.45	0	Novel
68	M	-	*PLBD2*	NM_173542.4	12	c.1576C>T	p.R526C	missense	comp. het.	0.05	0.000920308	25.8	VUS	−0.17	0	Novel
					12	c.1012C>T	p.R338W	missense		0.07	0.000272684	24.6	VUS	−0.17	0	
69	F	+	*DNAH3*	NM_001347886.2	16	c.2023G>T	p.V675F	missense	homoz.	0	0.0058627	23	LP	2.47	0	Known
70	M	-	*RECQL4*	NM_004260.4	8	c.2386G>A	p.E796K	missense	comp. het	0.01	3.40855 × 10^−5^	25.6	VUS	−6.29	0	Known
					8	c.3501C>T	p.I1167I	missense		0.01	6.8171 × 10^−5^	NA	VUS	−6.29	0	
71	M	-	*UBR4*	NM_020765.3	1	c.2551G>A	p.V851M	missense	comp. het	0.02	0.000852137	23	VUS	8.42	1	Known
					1	c.3137G>A	p.R1046Q	missense		0.0024	0.000136342	23.5	VUS	8.42	1	
72	F	+	*SNX21*	NM_033421.4	20	c.287C>T	p.A96V	missense	de novo	0	0	26	LP	−0.17	0	Novel
73	M	-	*FNDC1*	NM_032532.3	6	c.5047C>T	p.P1683S	missense	homoz.	0	0.000988479	25.2	LP	−0.57	0	Known
74	M	-	CNV identified													
75	M	-	CNV identified													
76	M	+	*TXNDC5*	NM_030810.5	6	c.625T>G	p.F209V	missense	homoz.	0	3.40855 × 10^−5^	29.6	LP	−1.09	0	Known
77	F	+	*TRIM73*	NM_198924.4	7	c.487C>T	p.R163*	nonsense	homoz.	0	0	35	P	3.23	0.93	Novel
			*MCM3*	NM_002388.6	6	c.2282A>G	p.Q761R	missense	homoz.	0	3.40855 × 10^−5^	24.6	LP	1.92	0	Novel
78	F	+	*ASGR2*	NM_001201352.2	17	c.532G>T	p.E178*	nonsense	de novo	0	0	36	P	0.28	0	Novel
79	M	+	*ATP13A5*	NM_198505.4	3	c.632A>G	p.Q211R	missense	de novo	0	0	26.1	LP	1.24	0	Novel
80	F	+	*TRIM15*	NM_033229.3	6	c.608A>G	p.D203G	missense	de novo	0	0	23.5	LP	−0.18	0	Novel
81	M	+	*ALG11*	NM_001004127.3	13	c.1184T>C	p.M395T	missense	homoz.	0	0	26	LP	1.11	0	Known
			*UBR1*	NM_174916.3	15	c.850G>C	p.E284Q	missense	homoz.	0	0.00190879	23.9	LP	3.29	0.96	Known
82	F	+	*RAP1GAP*	NM_002885.4	1	c.1429-864C>T	Intronic	intronic	homoz.	0	0	23.8	VUS	1.36	0.01	Novel
83	F	+	*CEP135*	NM_025009.5	4	c.3130G>C	p.E1044Q	missense	de novo	0	0	28	LP	−0.03	0	Known
			*APOL2*	NM_030882.4	22	c.943C>T	p.Q315*	nonsense	de novo	0	0	33	P	−0.6	0	Novel
			*HERC3*	NM_014606.3	4	c.38G>A	p.G13D	missense	de novo	0	0.000170427	27.1	LP	4.35	1	Novel
84	M	-	*DNAH17*	NM_173628.4	17	c.2121C>A	p.N707K	missense	comp. het.	0	3.40855 × 10^−5^	NA	LP	−2.01	0	Known
					17	c.12650T>C	p.I4217T	missense		0	0	26.9	LP	−2.01	0	
85	M	-	*DDR2*	NM_006182.4	1	c.473C>T	p.P158L	missense	de novo	0	0	25.8	LP	3.82	1	Known
86	F	+	*STRN4*	NM_013403.3	19	c.1610G>C	p.S537T	missense	homoz.	0	0.000272684	23.8	LP	2.14	0.84	Novel
87	M	+	*COL5A2*	NM_000393.5	2	c.4088G>T	p.G1363V	missense	homoz.	0	3.40901 × 10^−5^	32	LP	3.35	1	Known
88	M	+	*SPOUT1*	NM_016390.4	9	c.836T>C	p.F279S	missense	homoz.	0	0.000102256	27.5	LP	0.88	0	Novel
			*BNC1*	NM_001717.4	2	c.1697A>G	p.D566G	missense	homoz.	0	0.000579453	23.5	LP	2.25	1	Novel
89	F	+	CNV identified													
90	F	+	*SCN2A*	NM_001040142.2	2	c.532G>T	p.G178C	missense	de novo	0	0	28.3	LP	8.71	1	Known
91	M	+	*FMR1*	NM_002024.6	X	c.716C>T	p.A239V	missense	X-linked	0	0	25.3	LP	NA	NA	Known
92	M	+	*KDM6A*	NM_001291415.2	X	c.182G>C	p.R61T	missense	X-linked	0	0	22.6	LP	NA	NA	Known
93		-	*No SNV/CNV identified*													
94	M	+	*TMEM259*	NM_001033026.2	19	c.380A>G	p.Q127R	missense	de novo	0	0	25	LP	−2.09	0	Novel
95	M	-	*INTS6L*	NM_001351601.3	X	c.2584G>A	p.A862T	missense	X-linked	0	6.8171 × 10^−5^	22.2	LP	NA	NA	Novel
96	F	-	*GIPR*	NM_000164.4	19	c.1152G>T	p.Q384H	missense	comp. het.	0	0.000409026	36	LP	−1.18	0	Novel
					19	c.302G>A	p.R101H	missense		1.46	0.000647624	25.6	VUS	−1.18	0	
97	F	+	*SLC16A5*	NM_004695.4	17	c.380C>G	p.T127R	missense	de novo	0	0	23.9	LP	1.33	0	Novel
98	F	+	CNV identified													
99	M	+	*ARHGAP4*	NM_001666.5	X	c.1339T>C	p.Y447H	missense	X-linked	0	6.8171 × 10^−5^	24.9	LP	NA	NA	Novel
100	M	-	*GIPR*	NM_000164.4	19	c.1264C>T	p.Q422*	nonsense	de novo	0	0.000749932	36	P	−1.18	0	Novel
			*MAP3K5*	NM_005923.4	6	c.439T>C	p.Y147H	missense	de novo	0	0	32	LP	3.26	1	Novel
101	M	+	*CIP2A*	NM_020890.3	3	c.2303C>G	p.S768*	nonsense	de novo	0	0	41	P	−0.62	0	Novel
			*CLCNKA*	NM_004070.4	1	c.781+1G>A	splicing	splicing	de novo	0	3.40855 × 10^−5^	34	P	1.05	0	Known
102	M	+	*SAFB2*	NM_014649.3	19	c.1798G>A	p.D600N	missense	de novo	0	0	28.5	LP	−0.14	0.99	Novel
103	F	-	*ZNF827*	NM_001306215.2	4	c.1892A>T	p.D631V	missense	de novo	0	0	23.1	LP	2.95	1	Novel
104	F	+	*DENR*	NM_003677.5	12	c.434A>G	p.Q145R	missense	de novo	0	0	22.7	LP	1.6	0.92	Novel

A total of 116 unique genes were identified in 95 families, while 10 CNVs were detected in eight families. In family 93, neither SNV nor CNV was detected, and in 8 families, CNVs were detected, as shown in Table 3. Variants are denoted by their respective GenBank accession numbers. The term “NA” is used to signify “not available” when information on specific variants is absent. Of the 116 unique genes, 83 (marked in red) are considered highly likely to be causative for ASD based on criteria such as low prevalence, high-quality coverage, high CADD scores, significant pLI and Z-scores, and alignment with ACMG guidelines. These 83 genes were identified in 76 families, 6 genes found in 2 families each, and 1 gene identified in 3 families. Among the 83 genes highlighted in red, 48 are newly identified (novel), while 35 are known genes already associated with ASD or other NDDs. In the 56 consanguineous families, putative candidate genes were identified in 41 families. These included 41 homozygous recessive, 6 X-linked, and 6 de novo variants, with recessive genes accounting for 84% of the total. In the remaining 48 non-consanguineous families, we found 20 homozygous recessive, 7 X-linked, 10 de novo genes, with recessive genes comprising 56% of the findings. For de novo candidate genes, prioritization used to short-list was based on pLI scores above 0.9 and Z-score above 3. Additional metrics used include AF (allele frequency), pLI score, Z-score, and CADD score. QGP is Qatar Genome Program having allele frequencies from a control dataset of Qatari population. Chr. is chromosome and homoz. is homozygous recessive variant. Variants were classified using ACMG guidelines, which categorize genetic variants into benign, likely benign, uncertain significance (VUS), likely pathogenic (LP), and pathogenic (P) to assess their clinical significance.

**Table 3:** Ten heterozygous intragenic microCNVs identified in eight Omani ASD individuals.

Subject ID	Subject 19	Subject 25	Subject 52	Subject 54	Subject 74		Subject 75	Subject 89	Subject 98	
Sex	M	F	F	F	M		M	F	F	
CNV and genomic coordinates (hg38)	Chr16:6864585-6865758	Chr3:192671334-192672351	Chr19:4559368-4562532	Chr3:133414146-133418344	Chr10:54921983-54925066	Chr9:71289426-71290609	Chr17:45440911-45508556	Chr2:1521403-1522564	Chr10:26709200-26711140	Chr12:21165423-21166430
Type of CNV	heterozygous microdeletion	heterozygous microdeletion	heterozygous microdeletion	heterozygous microdeletion	heterozygous microdeletion	heterozygous microduplication	heterozygous microdeletion	heterozygous microdeletion	heterozygous microdeletion	heterozygous microdeletion
Size of CNV (bp)	1173	1017	3164	−4198	3083	1183	67645	1161	1940	1007
Cytoband	16p13.3	3q29	19p13.3	3q22.1	10q21.1	9q21.12	17q21.31	2p25.3	10p12.1	12p12.1
Genes involved	*RBFOX1* (NM_001142333)	*FGF12 * (NM_001377292)	*SEMA6B* (NM_032108)	*BFSP2* (NM_003571)	*PCDH15* (NM_001354404)	*TRPM3* (NM_001366141)	*PLEKHM1* (NM_014798)	*TPO* (NM_000547)	*PDSS1* (NM_001321978)	*SLCO1B1* (NM_006446)
Location	intron 3	intron 2	exon 1 and intron 1	intron 1	intron 3	intron 1	exon 6 (LRRC37A4P) exon 1-intron9 (PLEKHM1)	Intron 15	Intron 4, exon 5, intron 5	intron 2
Inheritance	de novo	de novo	de novo	de novo	de novo		de novo	de novo	de novo	
Disease linked	autism susceptibility 1; epilepsy, HGMD-71	developmental and epileptic encephalopathy, MIM 601513, HGMD-26	epilepsy, progressive myoclonic, MIM- 608873, HGMD-43	Cataract, MIM 603212, HGMD-13	Usher syndrome type 1; nonsyndromic genetic hearing loss, MIM 605514, HGMD-225	autosomal dominant non-syndromic intellectual disability, HGMD-24	osteopetrosis, MIM- 611466, PLEKHM1 HGMD-15	familial thyroid dyshormonogenesis, MIM 600044, HGMD-264	deafness–encephaloneuropathy–obesity-valvulopathy syndrome, MIM 607429, HGMD-19	rotor syndrome, MIM 604843, HGMD-51

Types and sizes of structural variants identified in Omani ASD individuals, along with their genomic coordinates (GRCh38/hg38) and associated genes, are detailed. These unbalanced genomic CNVs encompass at least one well-established gene linked to NDDs, supporting their potential pathogenicity. HGMD-71, 26, 43, 13, 225, 24, 15, 264, 19, and 51 are the number of variants reported in HGMD database on 27 November 2024 in the above-mentioned genes, respectively.

## Data Availability

Data are available upon request from corresponding authors.

## Supplementary Materials

The following supporting information can be downloaded at: https://www.mdpi.com/article/10.3390/ijms252413700/s1.

## Abbreviations

autism spectrum disorder (ASD), neurodevelopmental disorders (NDDs), intellectual disability (ID), next-generation sequencing (NGS), human gene mutation database (HGMD), genome sequencing (GS), single nucleotide variants (SNVs), copy number variants (CNVs), structural variants (SVs), Qatar-Genome Project (QGP), allele frequencies (AF).

## Web Resources

OMIM, https://www.omim.org/ (accessed on 1 August 2024).
HGMD, https://my.qiagendigitalinsights.com/bbp/view/hgmd/pro/all.php (accessed on 1 August 2024).
Genomics England NDD/autism panel genes https://panelapp.genomicsengland.co.uk/panels/285/ (accessed on 1 August 2024).
SFARI (Simons Foundation Autism Research Initiative) https://gene.sfari.org/database/human-gene/ (accessed on 1 August 2024).
