# Supplementary material for: Genetic Variant Analyses Identify Novel Candidate Autism Risk Genes from a Highly Consanguineous Cohort of 104 Families from Oman
Source: Int J Mol Sci. 2024 Dec 21;25(24):13700. doi: 10.3390/ijms252413700 (PMC11679916; doi:10.3390/ijms252413700)
Supplement: Supplementary file 1 [file ijms-3370267-supplementary.pdf › ijms-3370267-supplementary.pdf]

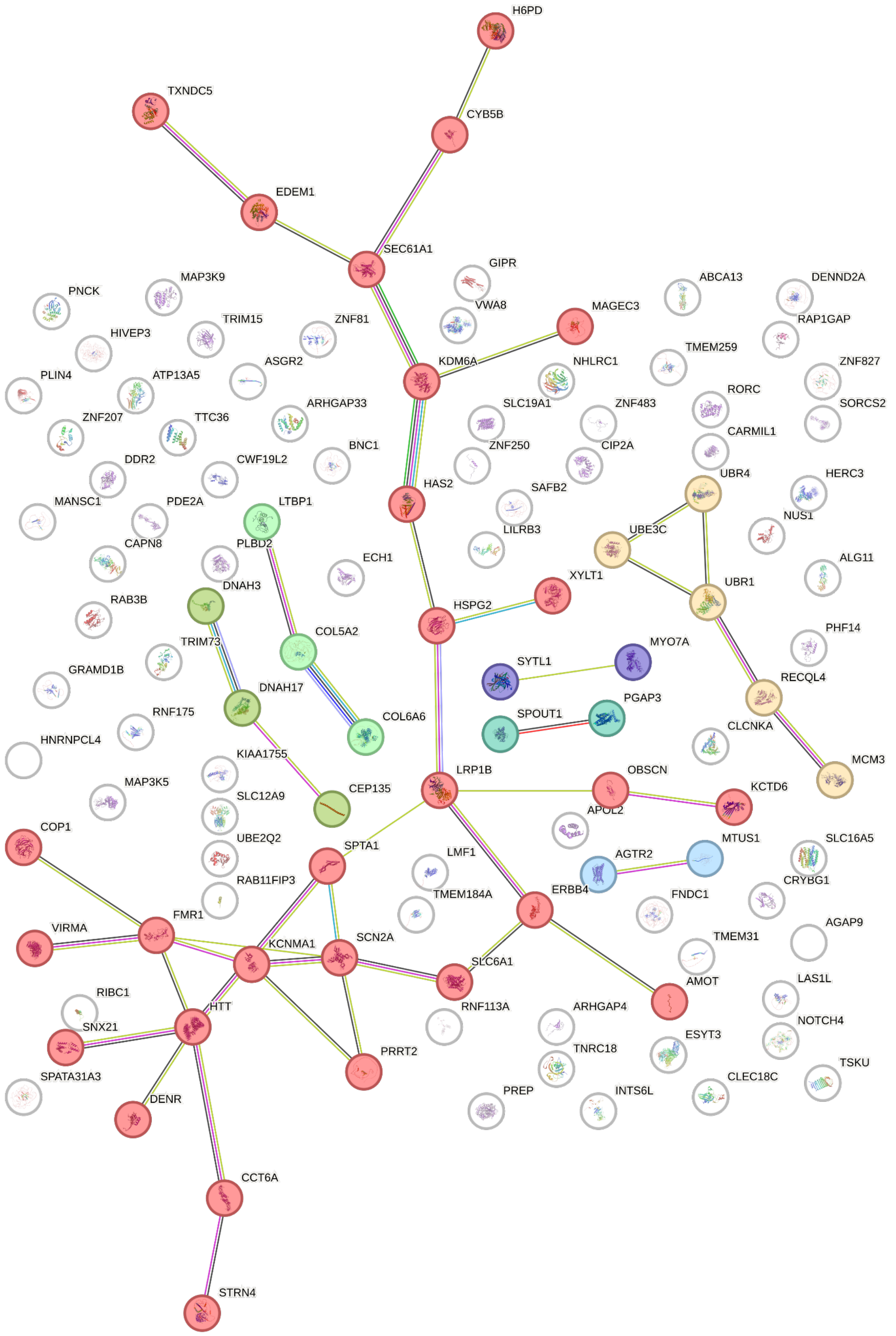

Suppl. Figure S1: STRING interaction enrichment analysis of the candidate genes identified two dominant enrichment networks represented by red (synaptic membranes) and yellow nodes (ubiquitin pathway).

Suppl. Fig. S1
